# Dietary Micronutrient Intake and Its Relationship with the Malnutrition–Inflammation–Frailty Complex in Patients Undergoing Peritoneal Dialysis

**DOI:** 10.3390/nu15234934

**Published:** 2023-11-27

**Authors:** Gordon Chun-Kau Chan, Jack Kit-Chung Ng, Phyllis Mei-Shan Cheng, Kai-Ming Chow, Cheuk-Chun Szeto, Philip Kam-Tao Li

**Affiliations:** 1 Carol & Richard Yu Peritoneal Dialysis Research Centre, The Chinese University of Hong Kong, Hong Kong, China; cck295@ha.org.hk (G.C.-K.C.); nkc426@ha.org.hk (J.K.-C.N.); cms439@ha.org.hk (P.M.-S.C.); ckm818@ha.org.hk (K.-M.C.); ccszeto@cuhk.edu.hk (C.-C.S.); 2 Department of Medicine & Therapeutics, Prince of Wales Hospital, Hong Kong, China; 3 Li Ka Shing Institute of Health Sciences (LiHS), Faculty of Medicine, The Chinese University of Hong Kong, Shatin, Hong Kong, China

**Keywords:** dietary pattern, zinc, malnutrition, inflammation, frailty, peritoneal dialysis

## Abstract

Background: The relationship between dietary patterns and the malnutrition–inflammation–frailty complex in patients undergoing peritoneal dialysis (PD) is currently unknown. Our objective was to measure dietary nutrient intake and evaluate its association with malnutrition, inflammation, and frailty. Methods: We prospectively recruited adult PD patients. We assessed their dietary nutrient intake using a food frequency questionnaire. Frailty, malnutrition, and inflammation were evaluated by validated Frailty Score (FQ), Subjective Global Assessment (SGA), and Malnutrition-Inflammation Score (MIS). Results: A total of 209 patients were recruited for the study. Among them, 89 patients (42.6%) had an insufficient protein intake, and 104 patients (49.8%) had an insufficient energy intake. Additionally, 127 subjects were identified as frail, characterized by being older (61.9 ± 9.5 vs. 55.6 ± 12.8, *p* < 0.001), malnourished (SGA: 21.0 ± 2.7 vs. 22.7 ± 3.1, *p* < 0.001), and having a high inflammation burden (MIS: 10.55 ± 3.72 vs. 7.18 ± 3.61, *p* < 0.001). There was a significant correlation between dietary zinc intake and body mass index (r = 0.31, *p* < 0.001), SGA (r = 0.22, *p* = 0.01), and MIS (r = −0.22, *p* = 0.01). In the multivariate model, a higher dietary zinc intake predicted a higher SGA (beta 0.03, *p* = 0.003) and lower FQ (beta −0.38, *p* < 0.001) and MIS (beta −0.14, *p* < 0.001), indicating a better nutrition, less frail and inflamed state. A higher dietary zinc intake was also associated with a lower odds of being frail (adjusted odds ratio 0.96, *p* = 0.009). Conclusion: Dietary inadequacy and micronutrient deficiency are common among the PD population. Dietary zinc intake is independently associated with an improved nutrition, physical condition, and reduced inflammatory state.

## 1. Background

Frailty is a condition characterized by vulnerability and decreased response to sudden stressors, which increases the likelihood of negative outcomes. In individuals with chronic kidney disease (CKD), frailty is associated with hospitalization, infection, cardiovascular events, complications related to dialysis, and death [1,2,3,4,5]. Sarcopenia is a common contributor to frailty in the general population, and it can be caused by malnutrition, leading to protein depletion in the body.

In individuals with CKD, the development of frailty is a more complex process. The retention of uremic toxins can cause the loss of appetite and fatigue. Dialysis therapy, specifically peritoneal dialysis (PD), can exacerbate the loss of appetite due to factors like abdominal distention and bloating [6]. The insufficient intake of protein and energy due to dietary restrictions, along with physical inactivity leads to protein-energy wasting, the breakdown of muscle tissue, and the development of sarcopenia. Additionally, high levels of circulating uremic toxins and the imbalance in acid–base homeostasis contribute to chronic inflammation and resistance to anabolic hormone action, resulting in catabolism and defective energy utilization.

The interaction between malnutrition, inflammation, and frailty is increasingly acknowledged as they often coexist and have a combined effect on patient outcomes [7]. To address malnutrition, a thorough nutritional assessment should be conducted, and medical nutritional therapy should be developed to optimize the patient’s nutrition status. This has the potential to improve outcomes such as hospitalization and mortality. However, there is currently no standardized guideline recommending the specific amount of replacement for each nutritional component or micronutrient. Additionally, the impact of individual micronutrient intake on frailty and its complex relationship with malnutrition and inflammation has not been formally assessed in the past. Our objective was to quantify the dietary nutrient intake in PD patients and evaluate its association with malnutrition, inflammation, and frailty.

## 2. Methods

We conducted a prospective single-center cohort study. Prevalent adult peritoneal dialysis (PD) patients were recruited from Prince of Wales Hospital, one of the two tertiary university-affiliated hospitals in Hong Kong from 1 June 2020 to 31 December 2022. The study protocol was approved by the Joint Chinese University of Hong Kong—New Territories East Cluster Clinical Research Ethics Committee (approval number: CRE-2019.715). The protocol was compliant to the Declaration of Helsinki, and all the participants provided written consent. Clinical data such as patient’s age, gender, primary diagnosis of renal disease, concomitant chronic medical illnesses; and laboratory parameters were obtained by chart review.

### 2.1. Dietary Nutrition Intake

Dietary nutrition intake was evaluated by a semi-quantitative food frequency questionnaire (FFQ) (Supplementary File S1), which has been validated and widely used in the past [8,9]. During the assessment, each subject recorded the weekly consumption of each food item with the assistance of well-trained personnel. These food items include grains, vegetables and beans, fruits, eggs, meat, fish and seafood, milk and beverages, snacks, and soup. The average nutrient intake was calculated and estimated by the nutrition table obtained from the McCance and Widdowson’s the composition of foods [10] and the Chinese Medical Sciences Institute [11]. Low dietary nutrient and vitamins intake were identified according to the KDOQI Clinical Practice Guideline for Nutrition in CKD [12], ESPEN guideline on Clinical nutrition in hospitalized patients with acute or chronic kidney disease [13], and The ASPEN Adult Nutrition Support Core Curriculum [14].

### 2.2. Nutrition and Inflammation

Nutrition was evaluated by the Subjective Global Assessment (SGA), which consists of several structured clinical parameters, namely weight loss, anorexia, and the loss of fat and muscle. SGA is a validated nutritional evaluation tool recommended by the KDOQI guidelines for use in dialysis patients [12].

Additionally, we used the Malnutrition-Inflammation score (MIS) [15] to measure the inflammatory burden. It consists of ten components under four major categories: (A) patients’ related medical history; (B) physical exam; (C) body mass index; and (D) laboratory parameters. The score ranges from 0 to 3 for each component, which makes up the total score of up to 30. A higher score indicates a higher inflammatory burden.

### 2.3. Frailty

Frailty was measured by a standardized validated questionnaire (FQ, Supplementary File S2). In short, the questionnaire contains 30 dichotomous questions, which comprehensively assesses various aspects of life quality, such as personal health, psychological and physical state, the need of assistance in different aspects of daily living, and mobility. The questionnaire was completed with the assistance of well-trained interviewers to ensure patients had sufficient understanding of each item. FQ has been extensively used by several published studies within the PD population. Each question scores 1 point, and the patients were classified as frail if they scored more than 6 points.

### 2.4. Statistical Analysis

Statistical analysis was performed by SPSS for Mac software version 29 (SPSS Inc., Chicago, IL, USA). Descriptive data were presented as mean ± standard deviation. Baseline clinical parameters were compared by Student’s *t*-test or Chi-square test as appropriate. Correlation analysis was performed by Pearson or Spearman’s rank correlation as appropriate. Linear and logistic regression models were constructed to identify predictors of outcomes by adding previously identified and potential confounding factors. Backward stepwise elimination was applied to remove insignificant variables. *p* < 0.05 was statistically significant. All probabilities were two-tailed.

## 3. Results

### 3.1. Clinical Characteristics

**Figure 1:**
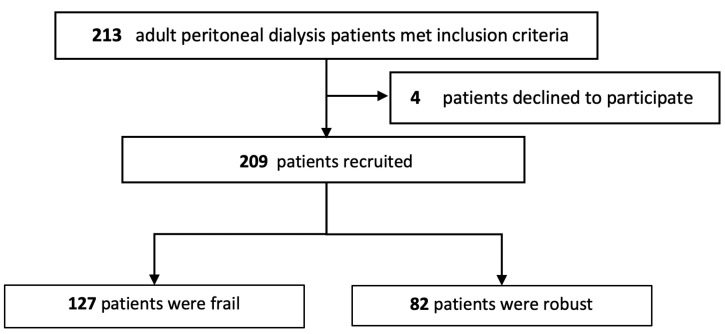
Flow chart.

A total of 213 adult patients undergoing PD who met the inclusion criteria underwent screening. Out of these, four patients declined to participate in the study, resulting in a recruitment of 209 patients. The study flow is summarized in Figure 1, and their baseline clinical and biochemical characteristics are presented in Table 1. The average age of the participants was 59.4 ± 11.3 years, with 104 (49.8%) being male.

Among them, 127 patients (59.6%) were frail. Frail subjects tend to be older (61.9 ± 9.5 vs. 55.6 ± 12.8, *p* < 0.001) and are more likely to have diabetes mellitus (52.8% vs. 32.9%, *p* = 0.005). A greater proportion of them underwent assisted PD (7.1% vs. 0%, *p* = 0.018).

### 3.2. Malnutrition–Inflammation–Frailty Complex

The body mass, nutritional, and inflammatory parameters of the subjects are summarized in Table 2. In summary, frail subjects have lower SGA scores across all individual components and the total score, indicating a more malnourished state. These subjects also have a high MIS score (*p* < 0.001), indicating a concomitant higher level of inflammation. This difference is primarily attributed to the disparity in sections A (patient’s related medical history) and B (physical examination) (*p* < 0.001 for both). In the correlation analysis, we found a strong correlation between body mass, malnutrition, inflammation, and frailty parameters (Table 3). In addition, we also found moderate and significant correlations between the MIS score and the neutrophil–lymphocyte ratio, the platelet–lymphocyte ratio, and C-reactive protein, indicating a connection between the MIS and other pro-inflammatory parameters (Table 3).

### 3.3. Dietary Nutrient Intake

The dietary nutrition intake of the patients is presented in Table 4. Out of the total, 89 patients (42.6%) had an insufficient protein intake, and 104 patients (49.8%) had an insufficient energy intake. Moreover, around 40% of the subjects had a low intake of vitamin B6 and B9, while 16.3% had low calcium intake and 19.6% had high phosphate intake. There is no correlation between the measured dietary intake and plasma levels of vitamins B9 (*p* = 0.2) and B12 (*p* = 0.4).

### 3.4. Relationship between Dietary Nutrient Intake and Malnutrition–Inflammation–Frailty Complex

There is a significant correlation between the intake of dietary zinc, folate, and B vitamins, namely vitamin B1 (thiamine), vitamin B2 (riboflavin), vitamin B3 (niacin), and vitamin B12 (cobalamin), with nutrition and inflammatory scores (Table 3). On the other hand, individuals who are frail have a different dietary pattern. When compared to individuals who are robust, frail individuals also had a noticeably lower average intake of vitamin D (10.2 ± 8.98 microgram vs. 15.6 ± 19.7 microgram, *p* = 0.04) and vitamin B7 (biotin) (89 ± 61 microgram vs. 128 ± 133 microgram, *p* = 0.027).

In the multivariate model, after adjusting for other dietary micronutrient intake, the intake of dietary zinc remains a significant independent determinant of FQ (*p* < 0.001), SGA (*p* = 0.003), and MIS (*p* < 0.001) (Table 5).

Moreover, a higher dietary zinc intake is linked to a lower probability of developing frailty, as shown in the logistic regression analysis of frailty predictors (adjusted odds ratio 0.96, 95% confidence interval 0.93–0.99, *p* = 0.009) (Supplementary Table S1). In the same model, the MIS score also serves as a significant predictor (adjusted odds ratio 1.24, 95% confidence interval 1.10–1.39, *p* < 0.001).

The relationship between micronutrients intake and components of frailty, nutritional and inflammatory markers are summarized in Supplementary Table S2. The intake of zinc (*p* = 0.02), vitamin D (*p* = 0.002), and calcium (*p* < 0.001) specifically significantly improves walking stability after accounting for confounding factors. Dietary zinc intake also improves muscle wasting in SGA (*p* = 0.002), and the medical history in by MIS (*p* = 0.004), whereas vitamin D intake affects the physical assessment of fat and muscle quantity during MIS assessment (*p* = 0.007).

## 4. Discussion

In this study, we analyzed the consumption of nutrients and micronutrients in a group of patients undergoing PD. Additionally, we examined and identified the significant correlation between nutrient intake, nutrition, inflammation, and physical health. Our findings indicate that patients with frailty exhibit a distinct pattern of nutrient consumption. Moreover, a higher zinc intake is linked to a lower risk of developing frailty and malnutrition. This study is the first to investigate the relationship between nutrient intake and frailty in PD patients, as well as to assess its connection with the triad of frailty, malnutrition, and inflammation. These findings suggest that micronutrients, particularly zinc, may play a role in the development of the malnutrition–inflammation–frailty complex.

The percentages of inadequate energy and protein intake in our cohort are significantly lower when compared to a previous Canadian study [16]. In that study, 94% and 71% of the 59 patients with advanced CKD had insufficient energy and protein intake. This difference can be explained by several factors. Firstly, our study had a larger sample size, recruiting almost four times as many participants. Additionally, our cohort had different subject characteristics, as only half of the patients in the previous study were on dialysis. Dialysis therapy itself and dialysis-related factors can contribute to catabolism and systemic inflammation. During dialysis, the resting energy expenditure significantly increases [17]. Furthermore, protein and vitamins may be lost during dialysis therapy [18]. However, patients undergoing dialysis receive more attentive support from nurses and dietitians. The peritoneal dialysate also contains a substantial amount of dextrose, providing additional calories and energy through peritoneal absorption. Therefore, it is important to consider that the impact of dietary nutritional values on outcomes in patients not undergoing dialysis should not be extrapolated to those undergoing dialysis.

Martín-del-Campo et al. [19] compared and reported on the intake of micronutrients based on the nutritional state in a group of patients undergoing PD. They found that malnourished individuals or those with high inflammation had a lower intake of dietary micronutrients. However, it is important to note that their results may be influenced by the fact that the amount of zinc intake could simply reflect the amount of food consumed. Additionally, the simultaneous intake of other proteins and micronutrients from foods containing zinc may also confound the results. To address this concern, we performed rigorous statistical adjustments by constructing several regression models. Our results indicate a persistent and significant association between zinc and other micronutrient intake with frailty, malnutrition, and inflammation, even after adjusting for potential confounding effects.

Contrary to the findings of Tseng PW et al. [20], who reported a positive and significant association between low protein intake and the development of frailty, we did not observe such an association in our cohort. This is consistent with a systemic meta-analysis conducted in the general population, which yielded conflicting results regarding the overall amount of protein intake and frailty development [21]. The impact of protein intake on frailty may vary depending on factors such as protein quality and the amount of protein distributed across meals. Therefore, it is recommended to consider protein source and ethnic differences in food cultural habits when examining this relationship.

In our current study, we present findings that show a positive relationship between the intake of zinc through our diet and improved nutrition, inflammation levels, and physical well-being. A higher intake of dietary zinc is specifically linked to a decrease in hospital and doctor visits, as well as improved mobility. However, it is important to note that zinc deficiency is quite common among individuals with CKD. In a study conducted with a Japanese cohort, more than half of the dialysis patients were found to have zinc deficiency, and the percentage is even higher among those undergoing PD [22]. A low dietary intake of zinc, in combination with protein restriction, often leads to zinc deficiency [23]. This creates a cycle as zinc deficiency can affect taste perception, further worsening malnutrition resulting from poor oral intake [24]. Zinc is an essential chemical element for the growth of muscles and bones [25]. Additionally, it plays a crucial role in reducing oxidative stress and slowing down the progression of metabolic diseases, such as diabetes and CKD, along with their associated complications [26]. Furthermore, zinc is necessary for a properly functioning immune system to defend against infections [27]. A study conducted by our center demonstrated that the presence of zinc-containing compounds in adipose tissue and serum can predict wasting and survival rates among dialysis patients [28]. Some research results also suggest that zinc supplementation plays a role in the growth of adipose tissue in animal models [29]. It is worth noting that advanced age, low BMI, and low albumin levels, which are significant predictors of zinc deficiency, overlap with the common characteristics of frailty. Since oral zinc supplementation has been shown to improve the control of anemia by increasing responsiveness to erythropoiesis-stimulating agents (ESAs) and reducing ESA dosage [30], it may potentially enhance muscle endurance and improve anemic control. However, it is important to note that high-dose zinc supplementation can lead to copper deficiency, which can also contribute to anemia [31]. The true benefits of zinc supplementation for nutritional and physical well-being require further research attention.

In addition to zinc, our results also indicate an association between frailty and dietary calcium and vitamin D. There are several explanations for this. Vitamin D promotes myogenesis and improves muscle contractility by activating the calcium-binding protein (calbindin-D9K), MAP kinase (MAPK), and phospholipase C (PLC) signaling cascade, which promote calcium influx into myocytes. Additionally, vitamin D improves energy homeostasis, reduces inflammation and intramuscular fatty infiltration, and increases muscle mass volume by reversing adipose browning [32]. Subjects with hypovitaminosis D often have difficulties in maintaining gait balance, leading to a higher chance of falls and associated injuries [33]. Multiple studies in animal models have demonstrated the effects of exogenous vitamin D therapy in attenuating protein energy wasting and improving muscle mass and function [32]. Observational studies have also shown that HD patients with vitamin D supplementation have better nutrition, less inflammation, and a lower fracture rate [34,35]. Large-scale randomized controlled trials should be conducted to study the effect of vitamin D supplementation, as prior small-scale studies have failed to demonstrate an improvement in terms of muscle strength, functional capacity, and health-related quality of life with such therapy [36,37].

Our study has some limitations. Firstly, it is a cross-sectional study, which means we can only detect associations but not establish causal relationships due to methodological flaws. Secondly, the recruitment was limited to patients attending hospital clinics, excluding those who are too frail or hospitalized. Thirdly, the small sample size reduces our ability to detect associations, although our sample size is larger than that of most published studies on dietary intake and nutritional state in the PD population. Instead of using biochemical parameters like serum albumin, C-reactive protein, and interleukin-6 as objective measures of inflammation, we used MIS. However, serum albumin has been criticized for its use as a marker of malnutrition and inflammation, as its levels can be affected by various conditions such as liver failure, burns, trauma, active infection, and protein-losing diseases (such as enteropathy, and urinary and dialysate protein loss). Additionally, MIS is closely correlated with serum CRP and IL-6, and has even better predictability for mortality in the dialysis population [38]. Lastly, there may be recall bias as the dietary questionnaire recorded dietary intake from past weeks.

## 5. Conclusions

In conclusion, we characterized the relationship between dietary micronutrient intake and the malnutrition–inflammation–frailty complex. Our findings indicate that higher zinc intake is associated with improved nutrition, physical state, and reduced inflammation. Further studies are needed to explore the potential benefits of zinc supplementation in promoting better nutrition and physical condition.

## Figures and Tables

**Table 1:** Clinical characteristics.

	Frail (*n* = 127)	Robust (*n* = 82)	*p*-Value
Age	61.9 ± 9.5	55.6 ± 12.8	*p* < 0.001 ^a^
Age older than 65	51 (40.2%)	19 (23.2%)	*p* = 0.03 ^b^
Male	63 (49.6%)	39 (47.6%)	*p* = 0.8 ^b^
Time on dialysis (months)	9.2 ± 50.7	25.5 ± 87.0	*p* = 0.09 ^a^
Primary cause of renal failure			*p* = 0.4 ^b^
- Diabetes mellitus	56 (44.1%)	25 (30.5%)	
- Hypertension	16 (12.6%)	9 (11%)	
- Glomerulonephritis	36 (28.3%)	33 (40.2%)	
- Urological cause	2 (1.6%)	2 (2.4%)	
- Polycystic kidney disease	5 (3.9%)	2 (2.4%)	
- Others	2 (1.6%)	2 (2.4%)	
- Unknown	10 (7.9%)	9 (11%)	
Comorbidities			
- Hypertension	117 (92.1%)	74 (90.2%)	*p* = 0.6 ^b^
- Diabetes mellitus	67 (52.8%)	27 (32.9%)	*p* = 0.005 ^b^
- Ischemic heart disease	15 (11.8%)	6 (7.3%)	*p* = 0.2 ^b^
- Cerebrovascular accident	12 (9.4%)	4 (4.9%)	*p* = 0.3 ^b^
Kt/V	1.78 ± 0.69	1.75 ± 0.44	*p* = 0.8 ^a^
Assisted peritoneal dialysis	9 (7.1%)	0 (0%)	*p* = 0.018 ^b^
Peritoneal dialysis (PD) modality			*p* = 0.4 ^b^
- Continuous ambulatory PD	109 (85.8%)	59 (72%)	
- Continuous cyclic PD	6 (4.7%)	4 (4.9%)	
- Nocturnal intermittent PD	12 (9.4%)	12 (14.6%)	
Systolic blood pressure (mmHg)	141 ± 22	140 ± 20	*p* = 0.7 ^a^
Diastolic blood pressure (mmHg)	77 ± 13	80 ± 12	*p* = 0.1 ^a^
Pulse rate (per minute)	79 ± 13	94 ± 11	*p* = 0.09 ^a^
Laboratory results			
- Hemoglobin (g/dL)	9.9 ± 1.5	10.0 ± 1.4	*p* = 0.6 ^a^
- White blood cell (10^9^/L)	7.3 ± 2.4	7.8 ± 2.7	*p* = 0.2 ^a^
- Neutrophil (10^9^/L)	5.2 ± 2.1	5.5 ± 2.4	*p* = 0.3 ^a^
- Lymphocyte (10^9^/L)	1.1 ± 0.4	1.3 ± 0.4	*p* = 0.045 ^a^
- Neutrophil–Lymphocyte ratio	5.5 ± 4.6	4.8 ± 2.4	*p* = 0.2 ^a^
- Platelet–Lymphocyte ratio	274 ± 316	232 ± 126	*p* = 0.3 ^a^
- C-reactive protein (mg/L)	1.8 ± 4.1	2.2 ± 5.9	*p* = 0.7 ^a^
- Total protein (g/L)	69.4 ± 6.8	67.6 ± 6.3	*p* = 0.07 ^a^
- Albumin (g/L)	28.6 ± 5.0	29.1 ± 4.5	*p* = 0.5 ^a^
- Calcium (mmol/L)	2.3 ± 0.2	2.3 ± 0.2	*p* = 0.03 ^a^
- Phosphate (mmol/L)	1.9 ± 0.5	2.0 ± 0.6	*p* = 0.07 ^a^
- Iron (microgram/L)	12.5 ± 5.6	14.5 ± 8.5	*p* = 0.049 ^a^
- Vitamin B12 (Cobalamin) (pmol/L)	115 ± 23	118 ± 16	*p* = 0.5 ^a^
- Vitamin B9 (Folate) (nmol/L)	23.9 ± 26.8	16.5 ± 9.1	*p* = 0.4 ^a^
- Cholesterol, total (mmol/L)	4.4 ± 1.7	4.5 ± 1.0	*p* = 0.6 ^a^
- Cholesterol, HDL-C (mmol/L)	1.2 ± 0.4	1.3 ± 0.4	*p* = 0.4 ^a^
- Cholesterol, LDL-C (mmol/L)	2.5 ± 1.6	2.6 ± 0.9	*p* = 0.6 ^a^
- Triglycerides (mmol/L)	1.8 ± 1.3	1.5 ± 0.9	*p* = 0.1 ^a^

Data are presented as mean ± standard deviation and compared by paired Student’s *t*-test ^a^ and chi-square test ^b^.

**Table 2:** Nutrition and inflammatory markers.

	Frail (*n* = 127)	Robust (*n* = 82)	*p*-Value
Body weight (kg)	62.2 ± 14.4	64.8 ± 17.6	*p* = 0.3
Body mass index (kg/m^2^)	23.9 ± 5.1	24.8 ± 9.4	*p* = 0.4
Waist circumference (cm)	92.6 ± 11.9	88.8 ± 16.3	*p* = 0.06
Hip circumference (cm)	95.2 ± 12.3	93.4 ± 13.2	*p* = 0.3
Normalized protein catabolic rate	1.78 ± 8.85	0.94 ± 0.17	*p* = 0.4
Subjective Global Assessment (SGA)			
- Weight loss	5.4 ± 0.8	5.7 ± 0.9	*p* = 0.003
- Anorexia	5.3 ± 0.7	5.7 ± 0.8	*p* = 0.001
- Loss of fat	5.3 ± 0.9	6.0 ± 1.8	*p* < 0.001
- Loss of muscle	5.0 ± 1.0	5.8 ± 1.1	*p* < 0.001
- Total score	21.0 ± 2.7	22.7 ± 3.1	*p* < 0.001
Malnutrition-Inflammation Score (MIS)			
- A—Patients’ related medical history	0.88 ± 0.43	0.48 ± 0.38	*p* < 0.001
- B—Physical exam	1.00 ± 0.70	0.52 ± 0.58	*p* < 0.001
- C—Body mass index	0.31 ± 0.70	0.29 ± 0.69	*p* = 0.9
- D—Laboratory parameters	1.91 ± 0.58	1.85 ± 0.92	*p* = 0.5
- Total score	10.55 ± 3.72	7.18 ± 3.61	*p* < 0.001

Data are presented as mean ± standard deviation and compared by paired Student’s *t*-test.

**Table 3:** Correlation between frailty, nutrition, inflammation, and dietary nutrient intake.

	BMI	SGA	MIS	FQ
SGA	r = 0.32	/	/	/
	*p* < 0.001			
MIS	r = −0.29	r = −0.70	/	/
	*p* < 0.001	*p* < 0.001		
FQ	r = −0.08	r = 0.48	r = 0.50	/
	*p* = 0.3	*p* < 0.001	*p* < 0.001	
C-reactive protein	r = 0.08	r = −0.09	r = 0.26	r = 0.20
	*p* = 0.4	*p* = 0.4	*p* = 0.006	*p* = 0.04
Neutrophil–lymphocyte ratio	r = −0.04	r = −0.19	r = 0.24	r = 0.03
	*p* = 0.6	*p* = 0.006	*p* < 0.001	*p* = 0.7
Platelet–lymphocyte ratio	r = −0.12	r = −0.14	r = 0.25	r = 0.01
	*p* = 0.08	*p* = 0.05	*p* < 0.001	*p* = 0.9
Zinc	r = 0.31	r = 0.22	r = −0.22	r = −0.67
	*p* < 0.001	*p* = 0.01	*p* = 0.01	*p* = 0.5
Vitamin B1 (Thiamine)	r = 0.24	r = 0.18	r = 0.18	r = −0.023
	*p* = 0.006	*p* = 0.045	*p* = 0.046	*p* = 0.8
Vitamin B2 (Riboflavin)	r = 0.25	r = 0.21	r = −0.20	r = −0.10
	*p* = 0.004	*p* = 0.015	*p* = 0.02	*p* = 0.3
Vitamin B9 (Folate)	r = 0.22	r = 0.20	r = −0.20	r = −0.12
	*p* = 0.01	*p* = 0.02	*p* = 0.028	*p* = 0.2
Vitamin B12 (Cobalamin)	r = 0.28	r = 0.24	r = −0.21	r = −0.11
	*p* < 0.001	*p* = 0.005	*p* = 0.019	*p* = 0.2

Data are compared by Spearman rank correlation coefficient. BMI, body mass index; SGA, Subjective Global Assessment; MIS, Malnutrition-Inflammation Score; FQ, Frailty Score.

**Table 4:** Weekly dietary micronutrients intake (per body weight in kilogram).

Dietary Micronutrients Intake (Unit)	
Protein (g)	8.2 ± 6.5
- Low protein intake	89 (42.6%)
Fat (g)	3.8 ± 3.4
Carbohydrates (g)	21.5 ± 13
Energy (kcal)	149.4 ± 99.4
- Low energy intake	104 (49.8%)
**Minerals**	
Calcium (mg)	2.5 ± 1.9
- Low calcium intake	34 (16.3%)
Phosphate (mg)	6.7 ± 3.7
- High phosphate intake	41 (19.6%)
Iron (mg)	1.4 ± 1.1
Zinc (mg)	1.3 ± 0.8
Copper (mg)	0.2 ± 0.1
**Fatty acids (FA)**	
Saturated FA (g)	0.9 ± 0.8
Polyunsaturated FA (PUFA) (g)	0.2 ± 0.2
Monosaturated FA (g)	1.1 ± 1.1
**Vitamins**	
Vitamin B1 (Thiamine) (mg)	0.1 ± 0.1
Vitamin B2 (Riboflavin) (mg)	0.1 ± 0.1
Vitamin B3 (Niacin) (mg)	2.1 ± 1.5
Vitamin B5 (Pantothenate) (mg)	0.3 ± 0.3
Vitamin B6 (Pyridoxine) (mg)	0.1 ± 0.1
- Low vitamin B6 intake	86 (41.1%)
Vitamin B7 (Biotin) (microgram)	1.8 ± 2
Vitamin B9 (Folate) (microgram)	15 ± 10.3
- Low vitamin B9 intake	84 (40.2%)
Vitamin B12 (Cobalamin) (microgram)	0.3 ± 0.3
Vitamin D (microgram)	0.2 ± 0.2
Vitamin K1 (microgram)	3.3 ± 6.1

Data are presented as mean ± standard deviation.

**Table 5:** Regression model on dietary micronutrient predictor of frailty, malnutrition, and inflammation.

	Body Weight		Subjective Global Assessment (SGA)		Malnutrition-Inflammation Score (MIS)		Frailty Score (FQ)	
	Beta (95% CI)	*p*-Value	Beta (95% CI)	*p*-Value	Beta (95% CI)	*p*-Value	Beta (95% CI)	*p*-Value
Zinc	0.41 (0.19–0.62)	*p* < 0.001	0.03 (0.009–0.05)	*p* = 0.003	−0.14 (−0.22–−0.06)	*p* < 0.001	−0.28 (−0.43–−0.12)	*p* < 0.001
Saturated fatty acid	0.38 (0.04–0.73)	*p* = 0.03					−0.08 (−0.13–−0.03)	*p* = 0.003
Vitamin D	0.77 (0.38–1.17)	*p* < 0.001			−0.13 (−0.24–0.01)	*p* = 0.03	−0.18 (−0.33–−0.02)	*p* = 0.03
Calcium							−0.003 (−0.005–−0.001)	*p* = 0.005

CI, confidence interval.

## Data Availability

The data generated in this study are available from the corresponding author upon reasonable request, subject to approval from the local authority.

## Supplementary Materials

The following supporting information can be downloaded at: https://www.mdpi.com/article/10.3390/nu15234934/s1, File S1: Food Frequency Questionnaire; File S2: Frailty Questionnaire; Table S1: Logistic regression analysis on the predictor of frailty; Table S2: Relationship between micronutrient intake and components of frailty, nutritional and inflammatory markers.

## References

[1] Chan G.C., Ng J.K., Chow K.M., Kwong V.W., Pang W.F., Cheng P.M., Law M.-C., Leung C.-B., Li P.K.-T., Szeto C.C. (2020). Interaction between central obesity and frailty on the clinical outcome of peritoneal dialysis patients. PLoS ONE.

[2] Chan G.C., Ng J.K., Chow K.M., Cheng P.M., Law M.C., Leung C.B., Li P.K.-T., Szeto C.-C. (2022). Polypharmacy Predicts Onset and Transition of Frailty, Malnutrition, and Adverse Outcomes in Peritoneal Dialysis Patient. J. Nutr. Health Aging.

[3] Chan G.C., Ng J.K., Chow K.M., Kwong V.W., Pang W.F., Cheng P.M., Law M.-C., Leung C.B., Li P.K.-T., Szeto C.-C. (2021). Progression in Physical Frailty in Peritoneal Dialysis Patients. Kidney Blood Press. Res..

[4] Chan G.C., Ng J.K., Chow K.M., Kwong V.W., Pang W.F., Cheng P.M., Law M.-C., Leung C.-B., Li P.K.-T., Szeto C.-C. (2021). Impact of frailty and its inter-relationship with lean tissue wasting and malnutrition on kidney transplant waitlist candidacy and delisting. Clin. Nutr..

[5] Chan G.C., Than W.H., Kwan B.C., Lai K.B., Chan R.C., Ng J.K., Chow K.-M., Cheng P.M.-S., Law M.-C., Leung C.-B. (2022). Adipose expression of miR-130b and miR-17-5p with wasting, cardiovascular event and mortality in advanced chronic kidney disease patients. Nephrol. Dial. Transpl..

[6] Kim S.M., Kang B.C., Kim H.J., Kyung M.S., Oh H.J., Kim J.H., Kwon O., Ryu D.R. (2020). Comparison of hemodialysis and peritoneal dialysis patients’ dietary behaviors. BMC Nephrol..

[7] Chan G.C., Fung W.W., Szeto C.C., Ng J.K. (2023). From MIA to FIFA: The vicious matrix of frailty, inflammation, fluid overload and atherosclerosis in peritoneal dialysis. Nephrology.

[8] Chan R., Leung J., Woo J. (2015). Dietary Patterns and Risk of Frailty in Chinese Community-Dwelling Older People in Hong Kong: A Prospective Cohort Study. Nutrients.

[9] Chan R.S.M., Yu B.W.M., Leung J., Lee J.S.W., Auyeung T.W., Kwok T., Woo J. (2019). How Dietary Patterns are Related to Inflammaging and Mortality in Community-Dwelling Older Chinese Adults in Hong Kong—A Prospective Analysis. J. Nutr. Health Aging.

[10] McCance R.A., Widdowson E.M. (2015). McCance and Widdowson’s the composition of foods. The Composition of Food.

[11] Yang Y.X. (2004). China Food Composition Table 2004 (Book 2).

[12] Ikizler T.A., Burrowes J.D., Byham-Gray L.D., Campbell K.L., Carrero J.J., Chan W., Fouque D., Friedman A.N., Ghaddar S., Goldstein-Fuchs D.J. (2020). KDOQI Clinical Practice Guideline for Nutrition in CKD: 2020 Update. Am. J. Kidney Dis..

[13] Fiaccadori E., Sabatino A., Barazzoni R., Carrero J.J., Cupisti A., De Waele E., Jonckheer J., Singer P., Cuerda C. (2021). ESPEN guideline on clinical nutrition in hospitalized patients with acute or chronic kidney disease. Clin. Nutr..

[14] ASPEN (2017). Adult Nutrition Support Core Curriculum.

[15] Kalantar-Zadeh K., Kopple J.D., Block G., Humphreys M.H. (2001). A malnutrition-inflammation score is correlated with morbidity and mortality in maintenance hemodialysis patients. Am. J. Kidney Dis..

[16] Sin D., Harasemiw O., Curtis S., Iman Y., Buenafe J., DaCosta J., Mollard R.C., Tangri N., Protudjer J.L.P., Mackay D. (2022). Dietary Patterns and Perceptions in Older Adults with Chronic Kidney Disease in the Canadian Frailty Observation and Interventions Trial (CanFIT): A Mixed-Methods Study. Can. J. Kidney Health Dis..

[17] Kaysen G.A., Greene T., Daugirdas J.T., Kimmel P.L., Schulman G.W., Toto R.D., Levin N.W., Yan G., Group H.S. (2003). Longitudinal and cross-sectional effects of C-reactive protein, equilibrated normalized protein catabolic rate, and serum bicarbonate on creatinine and albumin levels in dialysis patients. Am. J. Kidney Dis..

[18] Jankowska M., Lichodziejewska-Niemierko M., Rutkowski B., Debska-Slizien A., Malgorzewicz S. (2017). Water soluble vitamins and peritoneal dialysis—State of the art. Clin. Nutr..

[19] Martin-del-Campo F., Batis-Ruvalcaba C., Gonzalez-Espinoza L., Rojas-Campos E., Angel J.R., Ruiz N., Gonzalez J., Pazarin L., Cueto-Manzano A.M. (2012). Dietary micronutrient intake in peritoneal dialysis patients: Relationship with nutrition and inflammation status. Perit. Dial. Int..

[20] Tseng P.W., Lin T.Y., Hung S.C. Association of Frailty with Nutritional Status in CKD Patients. J. Ren. Nutr..

[21] Coelho-Junior H.J., Marzetti E., Picca A., Cesari M., Uchida M.C., Calvani R. (2020). Protein Intake and Frailty: A Matter of Quantity, Quality, and Timing. Nutrients.

[22] Shimizu S., Tei R., Okamura M., Takao N., Nakamura Y., Oguma H., Maruyama T., Takashima H., Abe M. (2020). Prevalence of Zinc Deficiency in Japanese Patients on Peritoneal Dialysis: Comparative Study in Patients on Hemodialysis. Nutrients.

[23] Mahajan S.K., Bowersox E.M., Rye D.L., Abu-Hamdan D.K., Prasad A.S., McDonald F.D., Biersack K.L. (1989). Factors underlying abnormal zinc metabolism in uremia. Kidney Int. Suppl..

[24] Tavares A., Mafra D., Leal V.O., Gama M.D.S., Vieira R., Brum I., Borges N.A., Silva A.A. (2021). Zinc Plasma Status and Sensory Perception in Nondialysis Chronic Kidney Disease Patients. J. Ren. Nutr..

[25] Hamza R.T., Hamed A.I., Sallam M.T. (2012). Effect of zinc supplementation on growth hormone-insulin growth factor axis in short Egyptian children with zinc deficiency. Ital. J. Pediatr..

[26] Gembillo G., Visconti L., Giuffrida A.E., Labbozzetta V., Peritore L., Lipari A., Calabrese V., Piccoli G.B., Torreggiani M., Siligato R. (2022). Role of Zinc in Diabetic Kidney Disease. Nutrients.

[27] Gammoh N.Z., Rink L. (2017). Zinc in Infection and Inflammation. Nutrients.

[28] Chan G.C., Than W.H., Kwan B.C., Lai K.B., Chan R.C., Teoh J.Y., Ng J.K., Chow K.M., Fung W.W., Cheng P.M. (2022). Adipose and serum zinc alpha-2-glycoprotein (ZAG) expressions predict longitudinal change of adiposity, wasting and predict survival in dialysis patients. Sci. Rep..

[29] Huang X., Jiang D., Zhu Y., Fang Z., Che L., Lin Y., Xu S., Li J., Huang C., Zou Y. (2017). Chronic High Dose Zinc Supplementation Induces Visceral Adipose Tissue Hypertrophy without Altering Body Weight in Mice. Nutrients.

[30] Kobayashi H., Abe M., Okada K., Tei R., Maruyama N., Kikuchi F., Higuchi T., Soma M. (2015). Oral zinc supplementation reduces the erythropoietin responsiveness index in patients on hemodialysis. Nutrients.

[31] Munie S., Pintavorn P. (2021). Erythropoietin-Resistant Anemia Secondary to Zinc-Induced Hypocupremia in a Hemodialysis Patient. Case Rep. Nephrol. Dial..

[32] Mak R.H., Querfeld U., Gonzalez A., Gunta S., Cheung W.W. (2021). Differential Effects of 25-Hydroxyvitamin D3 versus 1alpha 25-Dihydroxyvitamin D3 on Adipose Tissue Browning in CKD-Associated Cachexia. Cells.

[33] Wickstrom J.F., Sayles H.R., Graeff-Armas L.A., Yentes J.M. (2019). The Likelihood of Self-reporting Balance Problems in Those with Advanced Chronic Kidney Disease, Slow Gait Speed, or Low Vitamin D. J. Ren. Nutr..

[34] Matias P.J., Jorge C., Ferreira C., Borges M., Aires I., Amaral T., Gil C., Cortez J., Ferreira A. (2010). Cholecalciferol supplementation in hemodialysis patients: Effects on mineral metabolism, inflammation, and cardiac dimension parameters. Clin. J. Am. Soc. Nephrol..

[35] Meireles M.S., Kamimura M.A., Dalboni M.A., Giffoni de Carvalho J.T., Aoike D.T., Cuppari L. (2016). Effect of cholecalciferol on vitamin D-regulatory proteins in monocytes and on inflammatory markers in dialysis patients: A randomized controlled trial. Clin. Nutr..

[36] Hewitt N.A., O’Connor A.A., O’Shaughnessy D.V., Elder G.J. (2013). Effects of cholecalciferol on functional, biochemical, vascular, and quality of life outcomes in hemodialysis patients. Clin. J. Am. Soc. Nephrol..

[37] Singer R., Chacko B., Talaulikar G., Karpe K., Walters G. (2019). Placebo-controlled, randomized clinical trial of high-dose cholecalciferol in renal dialysis patients: Effect on muscle strength and quality of life. Clin. Kidney J..

[38] Rambod M., Bross R., Zitterkoph J., Benner D., Pithia J., Colman S., Kovesdy C.P., Kopple J.D., Kalantar-Zadeh K. (2009). Association of Malnutrition-Inflammation Score with quality of life and mortality in hemodialysis patients: A 5-year prospective cohort study. Am. J. Kidney Dis..

