# Supplementary material for: Dietary Micronutrient Intake and Its Relationship with the Malnutrition–Inflammation–Frailty Complex in Patients Undergoing Peritoneal Dialysis
Source: Nutrients. 2023 Nov 27;15(23):4934. doi: 10.3390/nu15234934 (PMC10707898; doi:10.3390/nu15234934)
Supplement: Supplementary file 1 [file nutrients-15-04934-s001.zip › Dietary FFQ Supplementary File S1.pdf]

Food Frequency Questionnaire

Grains

Type of Food	Code	How Often Within the Past Year?									How much each time?	Reference Portion
		Never	A Few Times per Year	Once per Month	2 – 3 Times per Month	Once per Week	Twice per Week	3 – 4 Times per Week	5 – 6 Times per Week	Every day		
Rice	274										bowls	1 bowl = 200g
Soft Rice	277										bowls	1 bowl = 100g
Congee	276										bowls	1 bowl = 100g
Wheat Noodles/Udon	270										bowls	1 bowl = 200g
Instant Noodles	272										bowls	1 bowl = 200g
Rice Vermicelli	280										bowls	1 bowl = 200g
Macaroni	269										bowls	1 bowl = 200g
Pasta	273										plates	1 plate = 100g
Oatmeal	286										bowls	1 bowl = 100g
Corn Flakes	311										boxes	1 box = 25g
Frosties	314										boxes	1 box = 25g
Chinese Steam Buns (Mann-Tau)	2023										pieces	1 piece = 50g
Plain rolls	254										pieces	1 piece = 70g
White Breads	251										slices	1 slice = 50g
White Breads (without edges)	251										slices	1 slice = 30g
Whole Wheat Breads	253										slices	1 slice = 50g
Whole Wheat Breads (no edges)	253										slices	1 slice = 30g
Sweet Rolls	255										pieces	1 piece = 70g

Vegetables & Beans

Type of Food	Code	How Often Within the Past Year?									How much each time?	Reference Portion
		Never	A Few Times per Year	Once per Month	2 – 3 Times per Month	Once per Week	Twice per Week	3 – 4 Times per Week	5 – 6 Times per Week	Every day		Never
Choy Sum	1202										plates	1 plate = 100g
Bok Choy	1201										plates	1 plate = 100g
Chinese Kale	1245										plates	1 plate = 100g
Broccoli	1207										plates	1 plate = 50g
Cauliflowers	1204										plates	1 plate =50g
Chinese Spinach	1211										plates	1 plate = 100g
Watercress	1237										plates	1 plate = 100g
Spinach	1270										plates	1 plate = 100g
Water Spinach	1209										plates	1 plate = 100g
Chinese Chives	1247										plates	1 plate = 100g
Pea Shoots	1242										plates	1 plate = 100g
Cabbages	1204										plates	1 plate = 100g
Celery Cabbages	1203										plates	1 plate = 100g
Lettuce	1205										plates	1 plate = 100g
Asparagus	1238										plates	1 plate =50g
Celery	1203										plates	1 plate =50g
Fresh Soybeans	1244										servings	1 serving = 50g
Tofu, Hard	1552										servings	1 serving = 50g
Tofu, Soft	1552										cubes	1 cube = 300g
Dried Tofu Sheets	1553										servings	1 serving = 50g
Deep Fried Tofu	1576										servings	2 pieces = 120g
Deep Fried Tofu Pockets	1554										pieces	1 piece = 16 g

Type of Food	Code	How Often Within the Past Year?									How much each time?	Reference Portion
		Never	A Few Times per Year	Once per Month	2 – 3 Times per Month	Once per Week	Twice per Week	3 – 4 Times per Week	5 – 6 Times per Week	Every day		
Tofu Skin	1556										servings	1 serving = 50g
Vegetarian Chicken	2651										servings	3 pieces = 50g
Wheat Gluten	284										servings	1 serving = 50g
Mungbean Sprouts	1222										plates	1 plate = 100g
Soybean Sprouts	1223										plates	1 plate = 100g
Baked Beans	1551										servings	1 serving = 50g
Red Bean (Dried)	1560										servings	1 serving = 25g
Black Eye Peas, dried	1558										servings	1 serving = 50g
Snap Beans	1243										plates	1 plate = 50g
Snow Peas	1230										plates	1 plate = 100g
Green Peas	1215										servings	1 serving = 50g
Broad Beans	1240										servings	1 serving = 50g
String Beans	1213										servings	1 serving = 50g
Onions	1226										servings	1 serving = 50g
Carrots	1216										servings	1 serving = 50g
Chinese Radish	1246										servings	1 serving = 50g
Sweet Potatoes	1251										servings	1 serving = 50g
Potatoes	1224										servings	1 serving = 50g
Pumpkins	1253										servings	1 serving = 100g
Lotus Roots	1250										servings	1 serving = 50g
Water Chestnuts	1249										servings	7 pieces = 50g
Bamboo Shoots	1241										servings	1 serving = 50g

Type of Food	Code	How Often Within the Past Year?									How much each time?	Reference Portion
		Never	A Few Times per Year	Once per Month	2 – 3 Times per Month	Once per Week	Twice per Week	3 – 4 Times per Week	5 – 6 Times per Week	Every day		
Bitter Melons	1248										servings	1 serving = 100g
Winter Melons	1212										servings	1 serving = 100g
Tomatoes	1221										servings	1 serving = 100g
Red Capsicum/pepper	1274										servings	1 serving = 100g
Green Capsicum	1273										servings	1 serving = 100g
Sweet Corns	1218										servings	1 serving = 50g
Canned Corns	1219										servings	1 serving = 50g
Angled Loofah	1252										servings	1 serving = 100g
Eggplants	1239										servings	1 serving = 100g
Fresh Mushrooms	1275										servings	10 pieces = 100g
Dried Mushrooms	1450										servings	12 pieces = 25g
Canned Mushroom	1453										servings	8 pieces = 100g<br>3 pieces = 50g
White Fungus	1458										servings	1 serving = 10g
Wood Fungus	1455										servings	1 serving = 10g
Black Moss	1454										servings	1 serving = 10g
Preserved Radish	1501										servings	6 slices = 50g
Preserved Vegetables	1503										tablespoons	1 tablespoon = 5 g
Preserved Greens	1502										tablespoons	1 tablespoon = 5 g
Chestnut	1566										pieces	1 piece = 10g
Cashew Nuts	1564										servings	1 serving = 35g
Peanut	1568										servings	1 serving = 25g

Fruits

Type of Food	Code	How Often Within the Past Year?									How much each time?	Reference Portion
		Never	A Few Times per Year	Once per Month	2 – 3 Times per Month	Once per Week	Twice per Week	3 – 4 Times per Week	5 – 6 Times per Week	Every day		
Oranges	1726										pieces	1 piece = 150g
Grapefruits	1706										pieces	1 piece = 150g
Apples	1701										pieces	1 piece = 150g
Pears	1717										pieces	1 piece = 180g
Bananas	1704										pieces	1 piece = 200g
Honeydew Melons	1714										servings	1 serving = 100g
Watermelon	1729										servings	1 serving = 100g
Pineapples	1724										slices	1 slice = 70g
Strawberries	1734										servings	4 pieces = 100g
Peaches	1721										pieces	1 piece = 200g
Mangos	1713										pieces	1 piece = 200g
Persimmons	1728										pieces	1 piece = 100g
Kiwi fruits	1731										pieces	1 piece = 75g
Apricots	1702										pieces	1 piece = 35g
Prunes	1720										servings	2 pieces = 100g
Grapes	3082										servings	10 pieces = 100g
Lychee	1711										servings	10 pieces = 100g
Longans	1712										servings	10 pieces = 30g
Cherries	1705										servings	10 pieces = 100g
Papayas	1716										servings	1 serving = 400g
Pomelo	1733										pieces	1 piece = 70g
Lemons	1710										pieces	1 piece = 100g

Type of Food	Code	How Often Within the Past Year?									How much each time?	Reference Portion
		Never	A Few Times per Year	Once per Month	2 – 3 Times per Month	Once per Week	Twice per Week	3 – 4 Times per Week	5 – 6 Times per Week	Every day		
Fruit Cocktail in Syrup	1740										servings	1 serving = 30g
Dried Apricot	1703										servings	10 pieces = 20g
Dried Prunes	1725										pieces	1 piece = 7g
Raisins	1709										boxes	1 small box = 40g
Dried Dates	1732										servings	15 pieces = 20g

Eggs

Type of Food	Code	How Often Within the Past Year?									How much each time?	Reference Portion
		Never	A Few Times per Year	Once per Month	2 – 3 Times per Month	Once per Week	Twice per Week	3 – 4 Times per Week	5 – 6 Times per Week	Every day		
Hard Boiled Eggs	1154										pieces	1 piece = 50g
Pan Fried Eggs	1155										pieces	1 piece = 50g
Eggs White	1152										pieces	1 piece = 50g
Century Eggs	1157										pieces	1 piece = 50g
Salted Duck Eggs	1156										pieces	1 piece = 50g
Quail Eggs	1158										pieces	1 piece = 10

Meats

Type of Food	Code	How Often Within the Past Year?									How much each time?	Reference Portion
		Never	A Few Times per Year	Once per Month	2 – 3 Times per Month	Once per Week	Twice per Week	3 – 4 Times per Week	5 – 6 Times per Week	Every day		
BBQ pork/Pork, Lean	580										servings	6 pieces = 50g
BBQ Pork, Lean & Fat	581										servings	5 pieces = 50g
Spare Ribs, Lean	569										servings	4 pieces = 50g
Spare Ribs, Lean & Fat	570										servings	4 pieces = 50g
Roast Pork, Lean	566										pieces	1 piece = 25g
Roast Pork, Lean & Fat (with 24% fat)	585										pieces	1 piece = 25g
Pork Chop, Pan Fried	567										pieces	1 piece = 100g
Beef Flank, Stir-Fried	650										servings	6 pieces = 50g
Beef Sirloin, Pan-Fried	551										Pieces	1 piece = 100g
Briskets	562										servings	4 pieces = 50g
Ox Tongue	561										servings	6 pieces = 50g
Chicken, with skin	602										servings	1 serving = 100g
Chicken, without Skin	601										servings	1 serving = 100g
Chicken Strips, Stir-Fried	612										servings	6 pieces = 50g
Chicken wing, mid section	604 (m)										pieces	1 piece = 30g
Chicken Wing, Whole	604 (w)										pieces	1 piece = 100g
Chicken Thigh	608										pieces	1 piece = 100g

Type of Food	Code	How Often Within the Past Year?									How much each time?	Reference Portion
		Never	A Few Times per Year	Once per Month	2 – 3 Times per Month	Once per Week	Twice per Week	3 – 4 Times per Week	5 – 6 Times per Week	Every day		
Roast Goose, with Skin	623										servings	1 serving = 100g
Roast Goose, no skin	624										servings	1 serving = 100g
Roast Duck, with Skin	620										servings	1 serving = 100g
Roast Duck, no skin	621										servings	1 serving = 100g
Lamb	640										servings	6 pieces = 50g
Baby Pigeons	630										whole	1 whole = 200g
Chicken Liver	610										slices	6 slices = 50g
Chicken Heart	617										slices	6 slices = 50g
Pig Liver	571										slices	6 slices = 50g
Pig Heart	587										slices	6 slices = 50g
Pig Kidneys	586										slices	6 slices = 50g
Beef Offal	2015										slices	6 slices = 50g
Pan Fried Hot Dogs	578										rolls	1 roll = 50g
Big Red Sausage	577										servings	6 slices = 50g
Chinese Sausage	574										rolls	1 roll = 50g
Chinese liver sausage	591										rolls	1 roll = 50g
Duck Thigh, Dried, Preserved & Salted	620										servings	1 serving = 50g
Pork, Dried, Preserved & Salted	589										servings	1 serving = 25g
Ham	575										servings	2 pieces = 50g
Canned luncheon Meat	572										servings	2 pieces = 50g
Hamburger Patties	552 (l)										pieces	1 piece = 70g

Fishes & Seafood

Type of Food	Code	How Often Within the Past Year?									How much each time?	Reference Portion
		Never	A Few Times per Year	Once per Month	2 – 3 Times per Month	Once per Week	Twice per Week	3 – 4 Times per Week	5 – 6 Times per Week	Every day		
Grass Fish	952										servings	1 serving = 200g
Big Head Fish	988										servings	1 serving = 100g
Mud Carp	985										servings	1 serving = 100g
Eel	975										servings	10 slices = 50g
Japanese eel	989										servings	10 slices = 50g
Blace	982										servings	1 serving = 100g
Golden Thread/Horse Head	953										servings	1 serving = 100g
Snake Head	984										servings	10 slices = 50g
Carp	981										servings	1 serving = 100g
Catfish	980										servings	1 serving = 100g
Grouper	954										servings	1 serving = 100g
Mackerel	991										servings	1 serving = 100g
Ribbon Fish	987										servings	1 serving = 100g
Big Eye Fish	992										servings	1 serving = 100g
Squid	955										servings	7 slices = 50g
Oysters	967										servings	6 pieces = 50g
Dried Oysters	968										servings	10 pieces = 50g
Prawns	964										servings	2 pieces = 25g
Crabs	969										servings	1 serving = 50g
Scallops/Dried Scallops	970										servings	3 pieces = 20g
Sea Cucumbers	976										servings	1 serving = 5g
Fish Balls	958										servings	5 pieces = 100g

Type of Food	Code	How Often Within the Past Year?									How much each time?	Reference Portion
		Never	A Few Times per Year	Once per Month	2 – 3 Times per Month	Once per Week	Twice per Week	3 – 4 Times per Week	5 – 6 Times per Week	Every day		
Fish Cakes	957										servings	4 slices = 50g
Cuttlefish	986										servings	7 slices = 50g
Mud Carp Fish Balls	985										servings	2 pieces = 50g
Canned Sardines	961										pieces	1 piece = 50g
Fried Dace with Black Bean Sauce	974										pieces	1piece = 50g
Tuna Fish	962										cans	1/3 can = 50g
Salted Preserved Fish	977										slices	1 slice = 5g
Jelly Fish	978										servings	1 serving = 50g
Salmons	990										pieces	5 pieces = 100g

Dairy Products & Beverages

Type of Food	Code	How Often Within the Past Year?									How much each time?	Reference Portion
		Never	A Few Times per Year	Once per Month	2 – 3 Times per Month	Once per Week	Twice per Week	3 – 4 Times per Week	5 – 6 Times per Week	Every day		
Whole Milk	76										cups	1 cup = 250ml
Skimmed Milk	79										cups	1 cup = 250ml
Chocolate Milk	78										cups	1 cup = 250ml
Whole Milk Powder	82										tablespoons	1 Tablespoon = 7g
Skimmed Milk Powder	83										tablespoons	1 Tablespoon = 7g
Condensed Milk	80										tablespoons	1 Tablespoon = 20g
Evaporated Milk	81										tablespoons	1 Tablespoon = 15g
Cheese	95										slices	1 slice = 20g
Whole Fat Yogurt	111										cups	1 cup = 150g
Low Fat Yogurt	112										cups	1 cup = 150g
Ice Cream	99 (c)										cups	1 cup = 134ml
Ice Cream Cone	99 (cone)										cones	1 cone = 70g
Milk Shake	2117										cups	1 cup = 300ml
Mayonnaise	2456										tablespoons	1 tablespoon = 15g
Chocolate Powder	2267										tablespoons	1 Tbsp = 5 g
Horlick	2268										tablespoons	1 Tbsp = 5 g
Ovaltine	2269										tablespoons	1 Tbsp = 5 g
Coca Cola	2251										cans	1 can = 350ml
Other sodas<br>Diet coke<br>Fanta Orange<br>Sprite	2252<br>2254<br>2253										cans	1 can = 350ml
Yakult	2260										bottles	1 bottle = 100g
Coconut Juice	2293										cups	1 cup = 250ml

Type of Food	Code	How Often Within the Past Year?									How much each time?	Reference Portion
		Never	A Few Times per Year	Once per Month	2 – 3 Times per Month	Once per Week	Twice per Week	3 – 4 Times per Week	5 – 6 Times per Week	Every day		
Vitasoy	2261 (s)										packs	1 pack = 250ml
Fresh Fruit Juices	2298										cups	1 cup = 250ml
Canned/Bottled Juices	2301										packs	1 pack = 250ml
Soy Milk	2292										cups	1 cup = 250ml
Sweetened Carton drinks	3016										packs	1 pack = 250ml
Chinese Tea	2282										cups	1 cup = 250ml
Green Tea	2272										cups	1 cup = 250ml
English Tea	2306										cups	1 cup = 250ml
Coffee	2273										cups	1 cup = 250ml
Wine	2276										cups	1 cup = 250ml
Spirits	2276										cups	1 cup = 250ml
Light Beer	2275										cups	1 cup = 250ml
Beer	2274										cups	1 cup = 250ml
Chinese wine	2277										cups	1 cup = 250ml
Mineral Water	2305										cups	1 cup = 250ml
Plain Water	W1										cups	1 cup = 250ml

Snacks

Type of Food	Code	How Often Within the Past Year?									How much each time?	Reference Portion
		Never	A Few Times per Year	Once per Month	2 – 3 Times per Month	Once per Week	Twice per Week	3 – 4 Times per Week	5 – 6 Times per Week	Every day		
Wonton	3066										pieces	1 piece = 20g
BBQ Pork Bun	2004										pieces	1 piece = 75g
Sweet Bun w/ Lotus Seed Paste/Egg Yolk Filling	2009										pieces	1 piece = 40g
Steamed Dim Sum	3060										pieces	1 piece = 20g
Deep Fried Dumplings	3064										pieces	1 piece = 35g
Steamed Cheung Fan (Steamed Rice Rolls)	3062										pieces	1 piece = 60g
Chinese Turnip Cake	2018										pieces	1 piece = 40g
Chicken Paw	2010										pieces	1 plate = 25g
"Yau-Char-Kwai" (Deep Fried Dough)	2020										pieces	1 piece = 70g
Pizza	2134										pieces	1 piece = 120g
Hamburger	2101										pieces	1 piece = 100g
Fish Burger	2109										pieces	1 piece = 140g
Chicken Nuggets	2108										pieces	1 piece = 20g
Hash Brown	2121										pieces	1 piece = 50g
Pork Pie	2042										pieces	1 piece = 100g
Apple Pie	2112										pieces	1 piece = 100g
Glazed Beef Jerky	564										pieces	3 pieces = 100g
Glazed Pork Jerky	579										pieces	3 pieces = 100g
Beef Floss	563										packs	1 pack = 25g
Pork Floss	579 (pack)										packs	1 pack = 25g
Dried Squid Strings	956										packs	1 pack = 20g

Type of Food	Code	How Often Within the Past Year?									How much each time?	Reference Portion
		Never	A Few Times per Year	Once per Month	2 – 3 Times per Month	Once per Week	Twice per Week	3 – 4 Times per Week	5 – 6 Times per Week	Every day		
Chinese Sweet Soup Desserts	2016										bowls	1 bowl = 200g
Tofu Fa	1555										bowls	1 bowl = 200g
Saltines/Cream Crackers	287										servings	3 pieces = 25g
Semi-sweet Biscuits	292										servings	2 pieces = 20g
Chocolate Coated Biscuits	295										pieces	3 pieces = 40g
Walnut Short Cakes	2040										pieces	1 piece = 150g
Milk Pudding	120										cups	1 cup = 120g
Egg Tart	302 (l)										pieces	1 piece = 60g
French Fries	2110										servings	1 serving = 100g
Potato Chips	2467										packs	1 small pack = 35g
Spongy Cake	301										pieces	1 piece = 70g
Pound Cake	300										slices	1 slice = 80g
Milk Chocolate	2461										pieces	1 piece = 10g
Candies	3017										pieces	2 pieces = 20g
Honey	2452										tablespoons	1 tablespoon = 20g
Jam	2453										tablespoons	1 tablespoon = 20g
Peanut Butter	2454										tablespoons	1 tablespoon = 15g
Corn Syrup	2457										tablespoons	1 tablespoon = 20g

Soups

Type of Food	Code	How Often Within the Past Year?									How much each time?	Reference Portion
		Never	A Few Times per Year	Once per Month	2 – 3 Times per Month	Once per Week	Twice per Week	3 – 4 Times per Week	5 – 6 Times per Week	Every day		
Herbs & Lean Pork Soup	J1										bowls	1 bowl = 200ml
Carrots & Green Turnips Soup	J1										bowls	1 bowl = 200ml
Watercress Soup	J1										bowls	1 bowl = 200ml
Radish & Mud Carp Dace Soup	J1										bowls	1 bowl = 200ml
Cabbages & Potatoes Soup	J1										bowls	1 bowl = 200ml
Peanuts Chicken Paws Soup	J1										bowls	1 bowl = 200ml
Split Peas & Peanut Soup	J1										bowls	1 bowl = 200ml
Hairy Melons & Octopus Soup	J1										bowls	1 bowl = 200ml
Cream of Chicken Soup	J1										bowls	1 bowl = 200ml
Vegetable & Tofu Soup	J1										bowls	1 bowl = 200ml
