# Supplementary material for: Dietary Micronutrient Intake and Its Relationship with the Malnutrition–Inflammation–Frailty Complex in Patients Undergoing Peritoneal Dialysis
Source: Nutrients. 2023 Nov 27;15(23):4934. doi: 10.3390/nu15234934 (PMC10707898; doi:10.3390/nu15234934)
Supplement: Supplementary file 1 [file nutrients-15-04934-s001.zip › Dietary FFQ Supplementary File S2.pdf]

Simplified Frailty Assessment Questionnaire

Name: ______________________ Date: _________________________

Question	Answer
Q1. Self perceived physical health not quite good/poor	Y / N
Q2. Doctor consultation in the past year 6+ times	Y / N
Q3. Hospital admission in the past year 2+ times	Y / N
Q4. No. of drug use 5+	Y / N
Q5. Weight loss for $\geq$5 lbs	Y / N
Q6. Headache in the past month	Y / N
Q7. Dizziness in the past month	Y / N
Q8. Have joint pain in the past month	Y / N
Q9. Feeling tired in the past month	Y / N
Q10. Falls in the past year: 1-2 times	Y / N
Q11: Falls in the past year: 3+ times	Y / N
Q12: Have chest pain while walking uphill or briskly	Y / N
Q13. Have chest pain while walking on level ground	Y / N
Q14. Cannot walk for 1 mile	Y / N
Q15. Cannot walk for 100 yards	Y / N
Q16. Wheezing or whistling in chest in the past year	Y / N
Q17. Wake up with a feeling of chest tightness in the past year	Y / N
Q18. Cough up phlegm for 3 consecutive months for 2 years	Y / N
Q19. Dependent in bathing	Y / N
Q20. Dependent in changing clothes	Y / N
Q21. Dependent in personal grooming	Y / N
Q22. Dependent in taking medicine	Y / N
Q23. Dependent in doing housework	Y / N
Q24. Dependent in doing shopping	Y / N
Q25. Need a walking aid usually	Y / N
Q26. Walking unsteadily or stagger	Y / N
Q27. Feeling your money / income is not enough	Y / N
Q28. Exercise less than 20 minutes each day	Y / N
Q29. Did not participate in social activity in the past month	Y / N
Q30. Feeling unhappy most of the time in the past week	Y / N

Total score: ___________
