# Supplementary material for: Dietary Micronutrient Intake and Its Relationship with the Malnutrition–Inflammation–Frailty Complex in Patients Undergoing Peritoneal Dialysis
Source: Nutrients. 2023 Nov 27;15(23):4934. doi: 10.3390/nu15234934 (PMC10707898; doi:10.3390/nu15234934)
Supplement: Supplementary file 1 [file nutrients-15-04934-s001.zip › Dietary FFQ Supplementary Materials.pdf]

Supplementary Materials

Supplementary File S1. Food Frequency Questionnaire (FFQ).

Supplementary File S2. Frailty Score (FQ).

Supplementary Table S1. Logistic regression analysis on the predictor of frailty

**Factor**	**Adjusted odds ratio (95% confidence interval)**	**P-value**
Dietary zinc intake	0.96 (0.93 – 0.99)	$p = 0.009$
Malnutrition-Inflammation Score	1.24 (1.10 – 1.39)	$p < 0.001$

Covariates added into the model: age, baseline SGA score, baseline MIS score, dietary energy, protein, zinc, saturated fatty acid, calcium, and vitamin D intake.

Supplementary Table S2. Relationship between micronutrient intake and components of frailty, nutritional and inflammatory markers.

(A) Zinc intake

**Frailty Score parameters**	**Odds ratio (95% confidence interval)**	**P-value**
- Q2: Doctor consultation in the past year 6+ times	0.66 (0.53 – 0.81)	p < 0.001
- Q3: Hospital admission in the past year 2+ times	0.88 (0.81 – 0.95)	p = 0.001
- Q25: Need a walking aid usually	0.79 (0.66 – 0.95)	p = 0.01
- Q26: Walking unsteadily or stagger	0.72 (0.54 – 0.95)	p = 0.02
- Q27: Feeling your money/income is not enough	0.81 (0.72 – 0.91)	p < 0.001
- Q30: Feeling unhappy most of the time in the past week	0.92 (0.87 – 0.97)	p = 0.004
**SGA parameters**		
- SGA muscle	0.04 (0.02 – 0.07)	p = 0.002
**MIS parameters**		
- MIS A	-0.01 (-0.02 - -0.003)	p = 0.004

(B) Vitamin D

**Frailty Score parameters**	**Odds ratio (95% confidence interval)**	**P-value**
- Q5: Weight loss =/> 5lbs in past year	0.53 (0.34 – 0.81)	p = 0.003
- Q7: Dizziness in the past month	0.57 (0.38 – 0.86)	p = 0.007
- Q25: Need a walking aid usually	0.63 (0.46 – 0.88)	p = 0.006
- Q26: Walking unsteadily or stagger	0.36 (0.19 – 0.69)	p = 0.002

- Q27: Feeling your money/income is not enough	0.79 (0.66 – 0.94)	p = 0.008
- Q29: Did not participate in social activity in the past month	0.08 (0.02 – 0.38)	p = 0.001
**MIS parameters**		
- MIS B	-0.03 (-0.05 – 0.007)	p = 0.007

(C) Calcium

**Frailty Score parameters**	**Odds ratio (95% confidence interval)**	**P-value**
- Q26: Walking unsteadily or stagger	0.99 (0.99 – 0.99)	p < 0.001
